# Incorporating Ecosystem Services into Water Resources Management—Tools, Policies, Promising Pathways

**DOI:** 10.1007/s00267-022-01640-9

**Published:** 2022-04-05

**Authors:** Derek Vollmer, Kremena Burkhard, Blal Adem Esmail, Paulina Guerrero, Nidhi Nagabhatla

**Affiliations:** 1 Moore Center for Science, Conservation International, Arlington, VA USA; 2 Institute for Environmental Planning, Leibniz University Hannover, Hannover, Germany; 3 Department of Landscape Ecology and Environmental Systems Analysis, Institute of Geoecology, Technische Universität Braunschweig, Braunschweig, Germany; 4 Institute of Geography, Ruhr University Bochum, Bochum, Germany; 5 United Nations University Institute on Comparative Regional Integration Studies (UNU CRIS), Bruges, Belgium; 6 School of Earth, Environment and Society, McMaster University, Hamilton, ON Canada

**Keywords:** Nature-based solutions, Blue and green infrastructure, IWRM, Water security, Ecosystem based adaptation, Adaptive water management

## Abstract

Ecosystems provide a range of services, including water purification, erosion prevention, and flood risk mitigation, that are important to water resource managers. But as a sector, water resources management has been slow to incorporate ecosystem protection and restoration, for a variety of reasons, although related concepts such as nature-based solutions and green infrastructure are gaining traction. We explain some of the existing challenges to wider uptake of the ecosystem services concept in water resources management and introduce some promising avenues for research and practice, elaborated in more detail through 12 papers, spanning five continents and a variety of contexts, which make up a Special Issue on “Incorporating Ecosystem Services into Water Resources Management”. Cross-cutting themes include (A) ecosystem services as a flexible concept to communicate with stakeholders; (B) participatory processes to involve stakeholders in research; (C) multiple values, and valuation methods, of water-related services; and (D) applications of decision-support tools. We conclude with a summary of research gaps and emphasize the importance of co-producing knowledge with decision makers and other stakeholders, in order to improve water resources management through the integration of ecosystem services.

## Introduction

Freshwater supply and other water services have been recognized as key contributions that ecosystems make to human well-being by proponents of the ecosystem services paradigm for nearly three decades now (De Groot et al. 2018; Finlayson et al. 2005; Vörösmarty et al. 2005). The list of water ecosystem services includes water provisioning (for human and non-human use), fisheries and other biotic materials, water purification, erosion prevention, flood protection, disease control, aquatic habitat provision, recreation, and other cultural services (Grizzetti et al. 2019; Nagabhatla and Metcalfe 2018). These services are often bundled in landscapes, meaning that a well-functioning forest or wetland ecosystem can provide multiple water (and other) ecosystem services (Vollmer et al. 2016), although in practice it may be more likely that water provisioning services are being optimized at the expense of other regulating and cultural services (Grizzetti et al. 2019).

The concept of water ecosystem services is well suited to help practitioners fulfill several ideals of another long-standing concept, namely integrated water resources management (IWRM) (Cook and Spray 2012), now enshrined as Target 6.5 of the UN’s Sustainable Development Goals. Specifically, IWRM calls for coordinated development of land and water resources, with attention to social benefits and equity, and sustaining ecosystems, all of which correspond well to focal points of the ecosystem services paradigm (Vlachopoulou et al. 2014; Vörösmarty et al. 2018). However, IWRM has long been criticized as being too top-down and idealized, thus rarely implemented (e.g., Giordano and Shah 2014). By contrast, an ecosystem services orientation could help in re-focusing IWRM away from prescriptive processes, and more toward people (beneficiaries) and local context, a “lighter” and more pragmatic approach to IWRM (Butterworth et al. 2010). Based on experience in landscape planning and management, key strengths of the ecosystem services concept in practice have been that it explicitly highlights relations between the state of natural assets (e.g., forests, wetlands) and human well-being, and that economic valuation and other established assessment methods resonate with decision makers’ motives and interests (von Haaren et al. 2019).

In parallel, there is also growing interest in ecosystem services-based approaches to enhance water security and resilience to climate change, especially in developing economies (e.g., Vogl et al. 2017, Adem Esmail and Geneletti 2020a, b; IPCC 2022). Concepts such as payments for ecosystem services, environmental economic accounting, nature-based solutions (NbS), blue-green infrastructure (BGI), and ecosystem-based adaptation have more recently emerged to further situate ecosystem services within a water resources management context. In their systematic review, Adem Esmail and Suleiman (2020a, b) identified 63 different terms used to refer to sustainable urban water management systems, indicating that—albeit with great diversity (or local specificity)—the principles of ecosystem services are being widely adapted into management concepts.

Yet implementation of the ecosystem services paradigm in the praxis of water resources management has been slow (Vlachopoulou et al. 2014; Souliotis and Voulvoulis 2021). Cook and Spray (2012) highlighted this implementation gap a decade ago, pointing to the inadequate integration of social and ethical factors alongside the environmental sciences agenda. They also note that ecosystem services researchers “must ask whether or how improved knowledge of human dependence on the physical environment is likely to address the self-interest that shapes environmental decision-making” (p 98). Harrison-Atlas et al. (2016), in their review of the literature on water ecosystem services, recommended more attention to actual decision contexts. Adem Esmail et al. (2017) emphasized how strategic ‘boundary work’ is crucial to facilitate co-production of knowledge in the water management sector—communicating, translating, and mediating issues related to services and tradeoffs. Hanna et al. (2018) noted a general lack of engagement with stakeholders when quantifying and valuing water ecosystem services, complicating efforts to make methods and results more relevant to decision making. After conducting a review of the many available ecosystem services mapping methods, Lavorel et al. (2017) recommend a focus on bridging the “biophysical realism gap” and to further improve the practice of quantifying ecosystem service supply. What is clear from these and other reviews is that there is a considerable amount of work in the research sphere on water ecosystem services, but a limited uptake in the resource management sphere.

This disconnect between research and practice is not unique to water ecosystem services and water resources management (Spyra et al. 2018; Longato et al. 2021), but there are some characteristics of each that make their integration challenging. Water ecosystem services are generally co-produced through a combination of interactions between natural, built, and human capital (Palomo et al. 2016; White et al. 2021; Adem Esmail and Geneletti 2020a, b). Identifying the specific contributions of the natural capital, that is, the additionality it provides to final services (Brauman 2015) is analytically challenging at the scale of hydrologic catchments because additional factors such as precipitation, slope, soil type, location and distance from both pressures and beneficiaries all significantly influence water ecosystem services (Sutherland et al. 2018). Even where we see integration of tools (e.g., hydrologic, hydraulic and ecosystem models) to provide this information for water resource managers (e.g., Adem Esmail and Geneletti 2017; Lin et al. 2021), there is still the need to reconcile the tradition of managing large centralized engineering structures with the distributed (and less certain) approach of managing networks of natural and semi-natural areas as green infrastructure that contribute synergically to the final services.

All these factors point to the need for more co-production of knowledge—that is, highly interactive goal-oriented research that is informed by context and draws on multiple stakeholders and forms of knowledge (Norström et al. 2020). Researchers tend to focus on the credibility of their methods to evaluate ecosystem services, but resource managers, including those related to the water sector also require methods that are cost-effective, meaningful, easily understood, scalable and applicable, implying the need for some give and take among all of these criteria in order to find suitable solutions (Olander et al. 2017). There is also an ongoing need for policies that encourage or at least support the integration of ecosystem services in water resources management. This includes explicitly recognizing ecosystem services in legislation (Liu et al. 2019), strengthening policy linkages across sectors (Carvalho et al. 2019), and reconciling spatial mismatches between sectoral policies and water ecosystem services (Qiu et al. 2017; Keiser et al. 2021). Again, research has an important role to play, identifying these policy needs but also highlighting promising examples where they exist.

These findings motivated our interest in preparing a special issue in *Environmental Management*. In mid-2020, we put out an open call for papers that could highlight the diversity of research taking place to apply the ecosystem services concept in water resource management. We encouraged critical analyses of methods, applications of decision-support tools, and case studies that demonstrate how context influences the way ecosystem services are interpreted and integrated into management. We also asked contributing authors to highlight factors that appear to be contributing to the success (or failure) of efforts to integrate ecosystem services. Finally, we encouraged authors to identify opportunities for further research that would help in either mainstreaming the use of the ecosystem services concept or improve its evidence base for a diverse global audience of practitioners.

## Incorporating Ecosystem Services into Water Resources Management

**Fig. 1:**
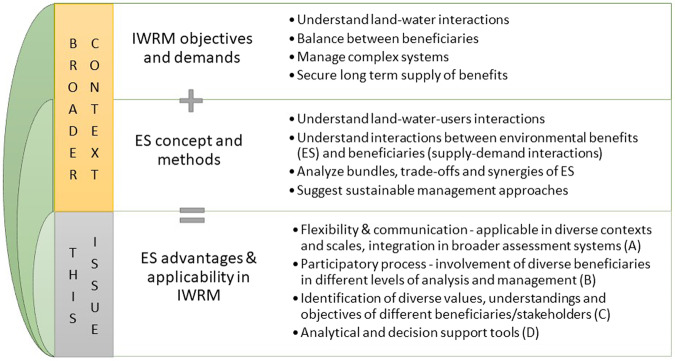
Themes addressed in this special issue—key characteristics of integrated water resource management (IWRM) and ecosystem services (ES) concepts and the prospects of their integration

This Special Issue brings together a set of articles representing a range of spatial scales, geographies, and topics in the area of ecosystem services and water management (Fig. 1). It has a strong emphasis on case studies, often co-produced with stakeholders and decision makers from the water resource management sector. The issue also highlights ecosystem services research from several regions that are underrepresented in the global literature, including sub-Saharan Africa and Southeast Asia. Geographic diversity is important not only to expand our understanding of ecosystem services performance in different hydro-climatic regimes, but also our understanding of the different socioeconomic and political contexts in which ecosystem services are being incorporated into water resource management. In the following sections, we expand on some of the themes that emerged from the articles in this special issue.

### Ecosystem Services as a Flexible Way to Connect with Stakeholders

While there is an ongoing debate about the need for standardization of definitions and approaches in ecosystem services assessments (Boerema et al. 2017, De Groot et al. 2018), one of the enduring strengths of ecosystem services as a concept is its flexibility (Hanna et al. 2018; Steger et al. 2018; Raheem and Schwarzmann 2021). The contributions in this special issue showcase how researchers and practitioners employ this flexibility to suit the data availability and informational needs of water resource managers around the world. Practicing integrated water resource management requires engaging with non-academic and non-technical stakeholders, and even key actors such as water resource engineers that may have limited to no experience in landscape ecology or ecohydrology and are more accustomed to thinking about water resources strictly in terms of quantity, quality and one-dimensional flow. The ecosystem services concept offers a way to discuss relevant topics (e.g., land-water interactions) from more of a lay perspective (Janssens de Bisthoven et al. 2021). However, being excessively rigid with ecosystem services terminology may undermine the intuitive nature of the concept (Marttunen et al. 2021); the goal when applying it to integrated water resource management is different from, for example, establishing national systems of ecosystem accounts, the latter requiring strict definitions and metrics.

Shaad et al. (2022) illustrate this with their application of ecosystem services indicators as part of a broader comprehensive assessment system—the Freshwater Health Index. Using a common framework for identifying and measuring water ecosystem services indicators, the authors demonstrate through case studies in China, Vietnam, Laos and Cambodia how the precise definitions (and data inputs) can be determined jointly with stakeholders, allowing for maximum flexibility. They suggest that ecosystem services indicators provide the first step toward deeper dialog with resource managers and other stakeholders on the provisioning, regulating, and cultural ecosystem services present within their watersheds, consistent with an approach that Olander et al. (2018) refer to as “benefit relevant indicators”.

The ecosystem services concept can also influence water resource management by way of legislation. Gomez-Betancur et al. (2021) provide an example of this from Latin America, where the first instance of a judicial decision referencing ecosystem services occurred in 2012, with the number of references (and referencing countries) steadily increasing year by year. The authors explore its implications through the lens of a landmark ruling by Colombia’s Constitutional Court regarding the Arroyo Bruno (Bruno River). In this case, the Court acknowledged that the Arroyo Bruno provides water, food, and various cultural and spiritual services that support an indigenous people’s (Wayuu) way of life. By ruling in favor of protecting the community’s rights to these ecosystem services, the Court expanded the discussion beyond water provision (which had been accounted for in the original environmental impact statement) and noted that certain services were not replaceable. Having this sort of legal ruling provides a precedent that can help shape subsequent policy and encourage additional communities to advocate for the protection of ecosystem services in their watersheds.

### Participatory Processes in Ecosystem Services Research

The ecosystem services concept is useful to engage a wide range of stakeholders in water resource management, but meaningful engagement requires facilitating their participation at various stages of research. When done well, this can lead to not only new, transformational knowledge, but also social learning, collective action, and improved competencies through capacity building (Schneider et al. 2019; Adem Esmail and Geneletti 2017). Marttunen et al. (2021) note that researchers should plan for an “appropriate intensity” of stakeholder engagement, based on the end goals of a project.

Stakeholder analysis is often an important starting point, to ensure representation and identify the likely roles that different stakeholder groups play in water resource management. Janssens de Bisthoven et al. (2021) elaborate on their analysis, the first step in preparation for developing a decision-support system for integrated water management in the Lake Manyara basin in Tanzania. They found that it is useful to specifically identify stakeholders who are directly dependent on resource extraction (water, fish, medicinal plants); they may have exceptionally high interest and can be locally influential on the ecology of the area but tend to have little or no political influence. This is an important finding, because it strengthens the rationale for engaging in a participatory process, while also offering specific guidelines on which stakeholder groups to approach and why, rather than a general imperative.

Bezerra et al. (2021) also relied substantially on stakeholder involvement in their research, holding a total of 10 workshops (in Brazil, Colombia, Peru, and Mexico) to introduce the Freshwater Health Index, conduct perception-based surveys, validate preliminary results, receive training, and communicate final results. Here, the authors report that stakeholders’ discussions around ecosystem services eventually revealed management blind spots. For example, in Peru, stakeholders had assigned a low weight (importance) to sediment regulation, but when that indicator received a low (poor) indicator score, it spurred discussion about the fact that forest conservation and restoration efforts may not currently be sufficient to safeguard this service, particularly in light of plans to expand hydropower in the region, since deforestation was occurring upstream of potential dam locations and excess sedimentation would decrease the lifespan and efficiency of these dams. This offers a practical example of how the ecosystem services concept (coupled with a participatory approach) can help unlock the potential of a more integrated approach to land and water management.

### Multiple Approaches to Valuing Ecosystem Services

Identification and prioritization of the values that ecosystems and their service have for different groups of stakeholders is arguably the most common way that the concept finds its way into resource management discussions, because it is an attempt to determine the ‘importance’ of ecosystem services, whether it is economic, social, or ecological (De Groot et al. 2010). While monetary valuation is still the most common approach, there are a range of perspectives on ecosystem services values and consequently many valid methods for discerning these values (Hubacek and Kronenberg 2013; Scholte et al. 2015). This Special Issue includes four case studies of ecosystem services valuation and showcases different ways to frame and elicit these values to guide water resource management decisions.

Tavárez et al. (2021) used contingent valuation methods, choice experiments and in-person interviews to estimate households’ willingness-to-pay (WTP) for gray and green interventions to increase water supply in rural Costa Rica. Specifically, they compared residents’ preferences for well construction, as a form of gray intervention, and reforestation, as a form of green intervention, aimed at alleviating water shortages. Interestingly, households were willing to pay a premium (25–34% of total WTP) to increase forest cover even if it did not provide additional benefits in terms of water supply. This is an important, policy-relevant demonstration of the added value of ecosystem protection and restoration—a full cost and benefit analysis of green and gray interventions should reflect the full range of values that beneficiaries associate with the green interventions.

Mulatu’s (2022) study showcases wetland ecosystem services valuation in South Sudan (the Sudd and Machar Marshes wetlands) to inform green infrastructure planning and development in the region and sustainable wetland management in the Nile Basin. They apply market price and benefit transfer approaches to value services, adjusting for local income and price differences and find the combined value is in excess of $2 billion annually. Their findings reiterate that the ecosystem services from these wetlands have benefits beyond the local communities and therefore a priority needs to be placed on addressing institutional weaknesses in natural resource management due to prolonged conflicts, instability and physical inaccessibility in the region. This underscores that a more integrated approach to management is not solved by simply drawing watershed boundaries; a network of local communities, civil society organizations, state and local government units, national ministries, and in this case international organizations are all key stakeholders, with varying levels of interest depending on the particular ecosystem service.

Morkūnė et al. (2021) demonstrate the importance of surveying different stakeholder groups, particularly when evaluating socio-cultural services. Their assessment of services in the Nemunas Delta region of Lithuania engaged farmers, birdwatchers, and scientists—three distinct groups with complementary knowledge but differing perspectives on the value of various services and actions that should be taken to safeguard services. Despite these differences, the assessment did highlight areas where values converged, such as water quality regulation, birdwatching, and other nature-based recreation. Understanding where opinions diverge (and converge) is useful in exploring policy options, to anticipate potential sources of conflict but also build on areas of agreement.

De Oliveira Rolo et al. (2021) evaluate a stream revitalization project in the city of São Paulo, Brazil, again focusing on local public perceptions as an indicator of ecosystem service values. In this case, ecosystem services were not explicitly part of the government’s stream revitalization program, so the assessment pointed out benefits that residents identified but that were not specifically considered in program design. In particular, residents highlighted improvements to water flow and quality, disease control, and recreation opportunities as a result of the revitalization efforts. As stream revitalization, ecological restoration, and even ecosystem-based adaptation projects grow in popularity, it will be critical to elicit this sort of information on stakeholders’ perceptions and values, to ensure that restoration projects are designed in a way to maximize benefits.

### Decision-Support Systems

Researchers have naturally gravitated toward decision-support tools and systems as a means of integrating information on ecosystem services, making it both accessible and understandable for decision makers without sacrificing credibility. Several decision-support tools already exist and are being used to operationalize ecosystem services in the water management sector (Grêt-Regamey et al. 2017). Nevertheless, existing tools are not always suitable to address the wide range of water management decision contexts (Brauman et al. 2021). For example, ecosystem service models (e.g., InVEST) were designed to work with a minimum level of data, sacrificing the precision and spatial/temporal resolution of more sophisticated hydrologic models (e.g., KINEROS, SWAT) (Vigerstol and Aukema 2011; Nedkov and Burkhard 2012; Boyanova et al. 2016). Still, data availability and technical capacity are widely considered to be barriers to greater uptake of these models, and there is a need to improve the representation of ecosystem processes in generating water ecosystem services (Lüke and Hack 2018). There has also been considerably less attention given to decision support regarding cultural ecosystem services (Plieninger et al. 2015; Grêt-Regamey et al. 2017) despite their importance in many freshwater systems.

In this issue, Martunnen et al. (2021) provide a review of studies that utilized the ecosystem services concept in multi-criteria decision analysis (MCDA) to support water management. They note that using the ecosystem services framework and classification in MCDA can strengthen decision makers’ recognition of the role of ecosystems in providing reliable water supplies, flood protection, and other benefits. In turn, the MCDA framework provides a standardized way to evaluate ecosystem services tradeoffs and to involve stakeholders in the evaluation (e.g., through weighting criteria). The authors conclude with some recommendations regarding the integration of ecosystem services and MCDA concepts, like visualizing assessment results and presenting a spectrum of preferences to highlight that both concepts invariably involve subjectivity. Making results more accessible and transparent is a way to move away from the technocratic, expert-dominated process that has often characterized IWRM.

Guerrero et al. (2022) combine geospatial identification of hydromorphological landscape units and ecosystem services assessment to support planning and implementation of NbS within the Lahn River floodplain landscape in Hesse, Germany. The delineation of hydromorphological landscape units through overlaying different landscape elements provides a more in-depth representation of diverse landscape functions, which are then used as indicators for the supply of ecosystem services. Thus, the capacity of the local landscapes to supply the analyzed ecosystem services (nitrogen retention, carbon storage, and outdoor recreation) are quantified based on the biophysical characteristics of the area and support the identification of opportunity spaces for NbS suited to the local environment. Through their biophysical assessment and mapping, ecosystem services can be used as a medium to facilitate understanding and communication of the effects and behavior of possible NbS and to identify optimal areas for their implementation. The authors urge for the values not to be taken as absolute but rather as a representation of existing patterns in the landscape that are based on quantitative data and can be used in the planning and implementation of NbS in management. In many cases, depending on the decision context, this level of information may not only be sufficient but also preferable to more complex modeling efforts, if it is considered transparent and practical (Brauman et al. 2021).

Bruen et al. (2022) combine biophysical and Bayesian Belief Network (BBN) models to better represent ecological variables and their response to physical and chemical stressors at the catchment scale. The BBN model integrates hydrologic and water chemistry modeled outputs, in-situ data, and expert knowledge to assess three ecosystem services (water quality regulation, wildlife appreciation, and recreational fishing) and their responses to scenarios of riparian buffer and livestock management. They test this approach on three catchments in Ireland and conclude that use of a BBN model helps to “bridge the gap” where formal models for specific ecosystem services do not exist, allows consideration of uncertainty, and offers flexibility to accommodate additional ecosystem services depending on the context. This modeling approach allows for relatively quick updating, which can help stakeholders engage with the information and better understand how changes in land or water management could impact the benefits they have identified.

## Future Research Needs

We conclude with some suggestions on research directions, reiterating that knowledge co-production is vital but requires genuine and focused commitment on the part of both researchers, resource managers and end-users (Chambers et al. 2021). The ecosystem services concept has proven useful in bringing additional disciplinary knowledge and stakeholder perspectives to bear on water resources management issues. Several of the articles in this special issue demonstrate that and provide examples of successful co-production in the water resources management sector and related fields. And as Marttunen et al. (2021) highlighted, it is important to explore ways to use the ecosystem services concept to structure water research, without letting it constrict thinking or cognitively overwhelm stakeholders.

A variety of terms and sub-concepts like NbS and BGI have evolved, stemming from different disciplinary areas and policy arenas, varying in terms of their primary focus (e.g., natural versus human systems) and specificity (e.g., from general principles to specific techniques) but often reflecting local understanding and interpretation (e.g., Fletcher et al. 2015). We see this as a logical and necessary step for a global and normative concept like ecosystem services to find its way into the generally local and context-dependent arena of water management. Acknowledging the plethora of sub-concepts, exploring their links and fostering exchanges between the respective communities remains imperative for incorporating ecosystem services into water—and more generally natural—resource management. NbS now have a globally recognized set of standards (IUCN 2020) but more work is needed to understand the “practical fit” of these concepts, i.e., understanding whether and how they are being translated into actionable programs that change actors’ behavior (Stevenson et al. 2021).

In line with the above argument, it is also important to consider the evolving pluralism and contextual constraints in integrating an ecosystem services approach. Take, for instance, Hamel and Tan’s review (2021) of BGI in Southeast Asia. Since much of the existing knowledge on BGI has been produced in the global North, in a limited range of climatic and socioeconomic contexts, it may not readily apply to vast regions of the globe now investing in BGI to help address water-related risks (see also Mulatu 2022). Bezerra et al. (2021) point out that, despite community dependence on inland fisheries in all three of their case study basins, there were no data and limited understanding of the importance of this service or how it was being impacted by water use and management decisions, an issue that is common particularly in the global South (Fluet-Chouinard et al. 2018). There is also a need for more research attention to riparian vegetation, river restoration, rice paddy agriculture (due to its unique hydraulic properties), and urban informal settlements when evaluating and designing interventions (Hamel and Tan 2021*;* De Oliveira Rolo et al. 2021).

There is an ongoing need to improve the quantitative representation of ecohydrology in ecosystem service assessments, particularly to capture a wider range of ecosystems and hydroclimates. Tavárez et al. (2021) demonstrate an approach to valuing hybrid green-gray water infrastructure but noted that many ecosystem services assessments rely on simplistic assumptions about the link between land cover and water provision, and that more empirical testing is needed, in a variety of contexts, to validate assumptions and models. Guerrero et al. (2022) reiterate the need to improve ecosystem services assessments by strengthening the physical and abiotic links to ecosystem processes and landscapes, rather than relying on land use or expert opinion. Credibility is particularly important when siting NbS, and so it is imperative to have models that adequately represent the ecohydrologic processes of interest (Brauman et al. 2021). But as Bezerra et al. (2021) highlight, technical capacity to apply these tools is frequently lacking in many parts of the world, and rectifying this should be a priority (e.g., through more capacity-building efforts) (Stevenson et al., 2021).

Ecosystem services research and related concepts, like NbS and BGI, indeed have much to contribute to water resources management. Realizing this potential requires an enduring commitment to interdisciplinary work—not just between ecologists and hydrologists, but among a wide range of natural, physical, and social sciences. Ecosystem services are co-produced, through interactions of ecological, technical, and social systems, and so it is important to understand under what conditions these services can be preserved or enhanced in the pursuit of more resilient and sustainable water management. Just as important is the involvement of end-users in the co-production of knowledge—this can take various forms but must be intentional and genuine, to ensure that the “right” research questions are being asked, and that methods and research design are oriented to deliver information that fits a particular water management context and needs. Finally, water-related policies should encourage an ecosystem services-oriented approach to management, with the caveat that this should not become a top-down prescription, but an invitation to experiment and explore.
